# The Sea and Its Dead

**Published:** 1900-06-02

**Authors:** 


### THE SEA AND ITS DEAD.

From reports received from various seaside resorts it would appear that the response made by the cattle trade to the restrictions imposed by sanitary authorities on the landing of diseased cattle has been the simple one of throwing the diseased carcases overboard. The result has been that, while the port sanitary authorities are saved the trouble of destroying this offal, the shores of our seaside resorts are scattered over with the entrails of diseased beasts. Truly a case of robbing Peter to pay Paul. Naturally dwellers in London protest against such desecration of their favourite holiday resorts just as farmers on the coast protest against diseased carcases being deposited, as one may say, at their very doors, and in protesting both are right. At the same time one may be allowed to suggest that London does not intervene in the matter with quite such clean hands as do the farmers, for London, unfortunately, is a grievous sinner in regard to this very matter of throwing things overboard. Every day throughout the year great steamships, specially constructed for the purpose, carry thousands of tons of London refuse in the form of sludge, which they calmly "tip" into the sea off the Nore, leaving fishes to deal with it as they find best. The evil produced by the millions of millions of microbes is no doubt less obvious than that caused by a few stones of rotting entrails. It is, however, of the same nature, and we cannot but think that the time is approaching when we shall no longer be content with such a primitive method of getting rid of nuisances, whether they take the form of sewage, sludge, or diseased cattle, as by casting them into the sea.

